# Preparation of Nanowire like WSe_2_-Graphene Nanocomposite for Photocatalytic Reduction of CO_2_ into CH_3_OH with the Presence of Sacrificial Agents

**DOI:** 10.1038/s41598-017-02075-7

**Published:** 2017-05-12

**Authors:** Asghar Ali, Won-Chun Oh

**Affiliations:** Department of Advanced Materials Science & Engineering, Hanseo University, Seosan, 31962 Korea

## Abstract

A nanowire-like WSe_2_-graphene catalyst was prepared via ultra-sonication and was tested in terms of the photocatalytic reduction of CO_2_ into CH_3_OH under irradiation with UV/visible light. The prepared nano-composite was further characterized via XRD, SEM, TEM, Raman and XPS. The photocurrent analysis was further tested for its photocatalytic reduction of CO_2_ using gas chromatography (GCMS-QP2010 SE). To further improve the the photo-catalytic efficiency, a sacrificial agent (Na_2_S/Na_2_SO_3_) was added to the WSe_2_-graphene nanocomposite and was found to improve the photo-catalytic efficiency, with the methanol yield reaching 5.0278 µmol g^−1^h^−1^. Our present work provides a convenient way to prepare nanomaterials various morphologies that have future applications for environmental remediation.

## Introduction

The increase in the amount of the CO_2_ emissions in the environment has reached an alarming level, greatly contributing to global warming^1^, and in the last few decades, the shortage of fossil fuel resources has resulted in an increased demand for energy production from different sources, including photovoltaic and photocatalytic H_2_ production. One such technology is photocatalytic CO_2_ reduction, which is a most valuable approach to overcome both global environmental and energy problems due its low cost, clean energy production, and environmental friendliness^2, 3^. In particular, the photocatalytic reduction of CO_2_ can convert noxious gasses (carbon dioxide) into profitable solar fuels, i.e., CO, HCHO, CH_3_OH, and CH_4_ by using solar energy^4, 5^. To date, many photocatalysts such as TiO_2_, CdS, g-C_3_N_4_, ZnO and Bi_2_WO_6_ have been shown to achieve the photo reduction of CO_2_^6–10^. Unfortunately, there are limits to the practical applicability of these semiconductors because the efficiency of the CO_2_ conversion into useful products is very low due to many factors of the CO_2_ reduction process, e.g., band gap tuning, charge carrier recombination, and light utilization^11^. Due to the wide band gap energy, most materials are unable to absorb visible light, and on the other hand, small band gap semiconductors exhibit a fast recombination phenomenon. To overcome this and to find an achievable process, highly efficient, low cost transition metal dichalcogenides (TMDC) have been investigated^12–15^. The generalized formula for TMDs is MX_2_, where M = transition metal element of group 4, 5 or 6 and X represents a chalcogen. Tungsten diselenide (WSe_2_) is a semiconductor that belongs to the TMDC family. Its structure consists of Selenium and tungsten held together by a weak van der Waals force that allow the material to be exfoliated into monolayers^16, 17^. Moreover WSe_2_ is a layered semiconductor with a small band gap of approximately 1.6 eV^18^. It has unique electrical transport performance, and its use was recently reported in many advanced energy storage applications, such as in superconductors^19^, lithium ion batteries^20^, photodetectors^21^, and photocatalytic hydrogen production^22^ in solar cells. Furthermore, Li, Yanguang *et al*. and Merki, Daniel *et al*. indicated that both the theoretical calculation and experimental results for the hydrogen evolution reaction of transition metal dichalcogenide (WSe_2_) materials appears to be from active edge sites that play an important role in hydrogen evolution reaction catalysis^23, 24^. However, due to the intrinsic interlayer *Van der Waals* attraction, the layered TMDC materials can be easily re-stacked as a result of reducing the active edge sites. Therefore, to improve photocatalyitc activity, the TMDC (WSe_2_) materials are combined with 2D carbon material such as graphene.

Graphene has unique properties, including a large surface area, good conductivity, and high flexibility, and it is used in high-performance energy storage devices^25, 26^. Therefore, coupling with a photocatalyst may improve the photocatalytic reduction efficiency of CO_2_ to fulfill the practical requirements. Liange *et al*. first described that coupling with a semiconductor is one of the best ways to boost the photocatalytic reduction efficiency of CO_2_^27^. Recently, graphene coupled with TMDCs (WSe_2_) was reported for the hydrogen evolution reaction (HER)^28^, high-performance oxygen reduction reaction (ORR)^29^ and in a superconductor^30^. However, the WSe_2_ chalcogenide family has not yet been analyzed for the photocatalytic reduction activity of CO_2_. In the present study, we report on WSe_2_-graphene nano composites prepared via ultra-sonication. The prepared samples (WSe_2_-graphene) were used for CO_2_ reduction, and the results exhibit a high efficiency for photo catalytic CO_2_ conversion under UV and visible light irradiation.

**Figure 1:**
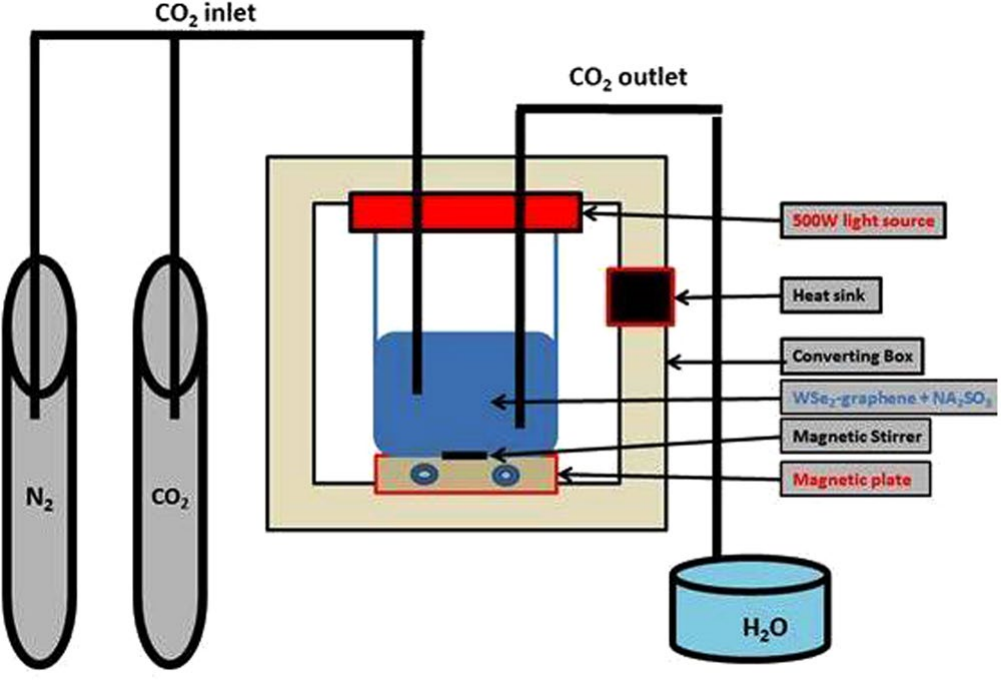
The schematic diagram of CO_2_ reduction device.

## Results and Discussion

### Characterization of catalytic materials

**Figure 2:**
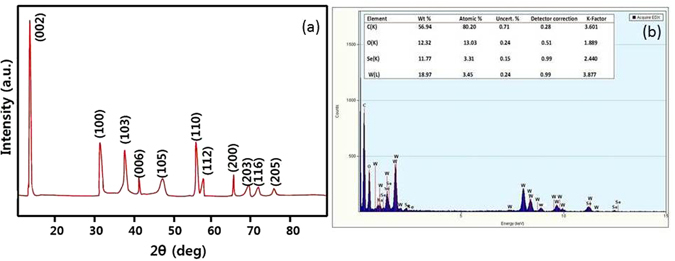
(**a**) XRD pattern and (**b**) EDX of WSe_2_-graphene composite.

The XRD patterns of the WSe_2_-graphene nanocomposites is shown in Fig. 2(a), the diffraction peaks at 13.54, 32.50, 38.45, 41.86, 47.75, 56.25, 58.10, 66.77, 69.45, 72.55 and 76.78° correspond to (002), (100), (103), (006), (105), (112), (200), (203), (116) and (205) directions, respectively (a = b = 0.329 nm, c = 1.298 nm, JCPDS PDF# 00-38-1388)^28, 29^. However, no other diffraction peaks form any other chemical species were observed in the prepared sample. For the GO analysis, a sharp peak (002) occurs at 13.44°, revealing that the graphite power was converted into graphene oxide by expanding the d-spacing from 3.5 to 6.78 Å^31, 32^. Moreover, the results indicate that the XRD signals of GO are very weak and are overlapped with those of WSe_2_-graphene at 13.54°. Therefore, the measurements were unable to detect the weaker diffraction peaks of GO^33^. Further EDX spectra confirm the presence of the main elements in the catalyst composites. The Fig. 2(b) shows the presence of the prime elements C, O, W, and Se, The C elemental peak is derived from graphene sheet, and W, Se and O are the precursor material.

**Figure 3:**
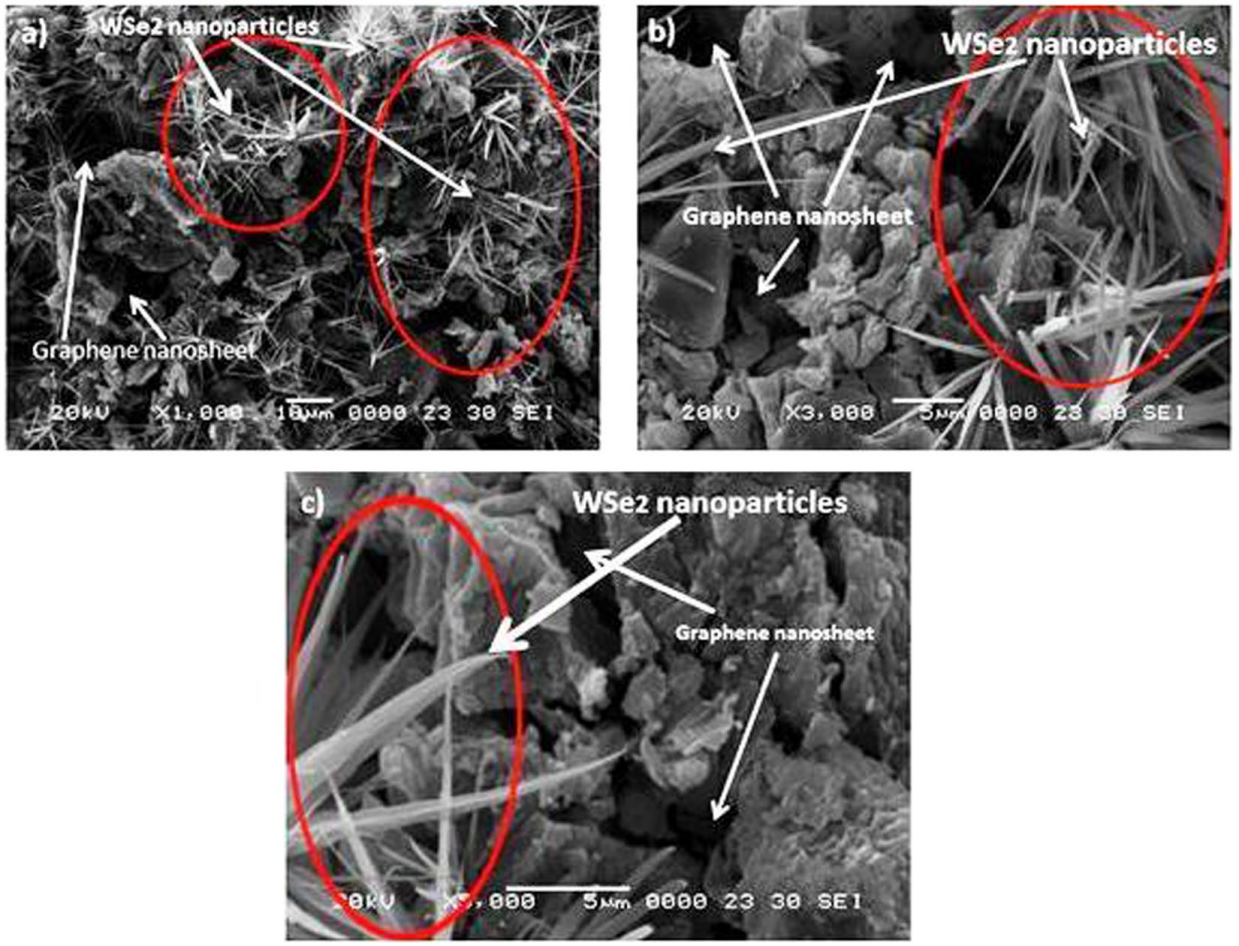
SEM imges of WSe_2_-graphene composite with different mignification.

**Figure 4:**
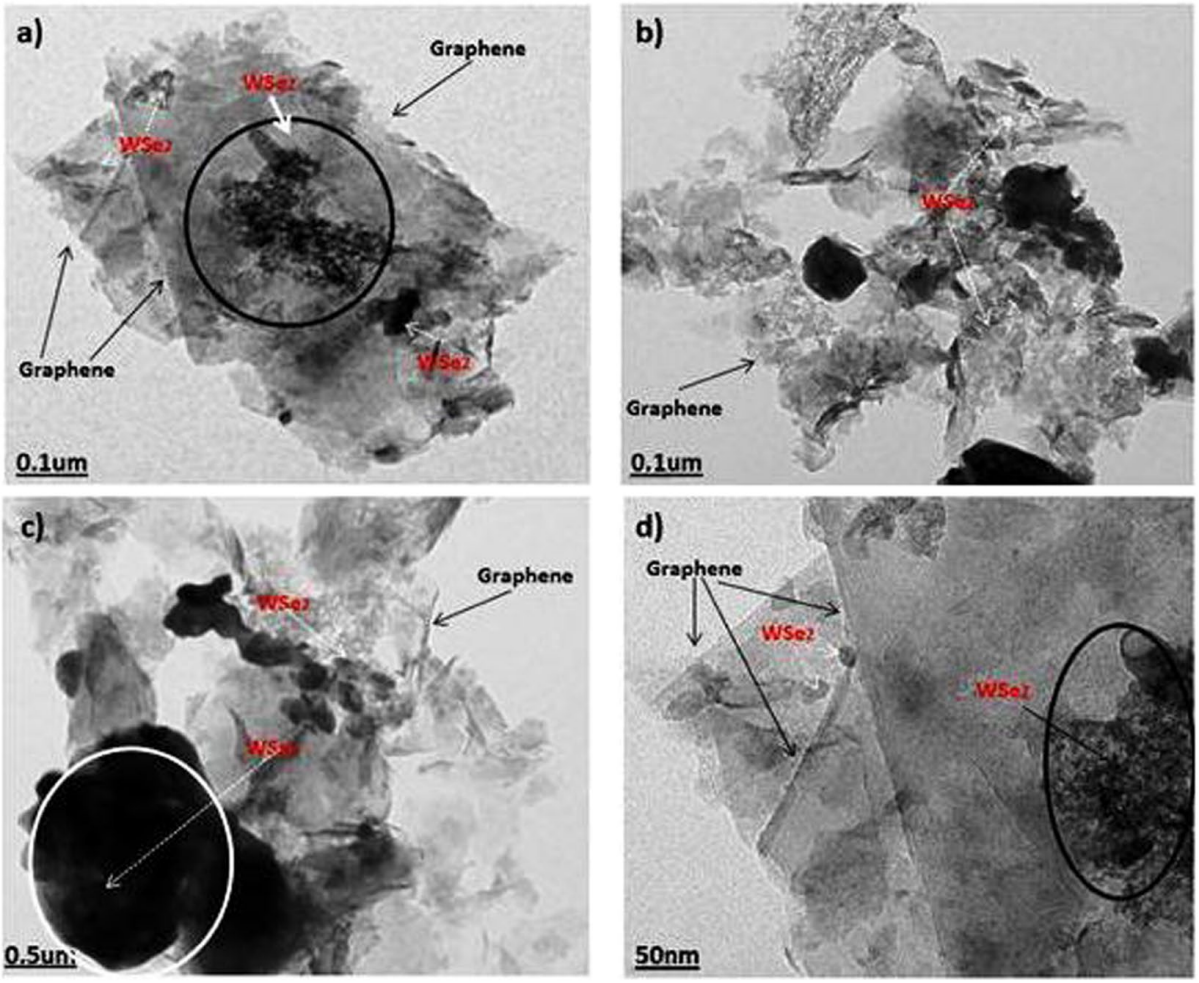
TEM image of WSe_2_-graphene.

SEM observations were carried out to investigate the surface and structural morphology. Figure 3 shows the SEM images of WSe_2_-graphene at different magnifications. Figure 3(a) shows the wire-like and nanoscale WSe_2_ morphology composed of interlaced and ultrathin nanosheets uniformly dispersed on the graphene nanosheet. Figure 3(b) and (c) show that graphene has an irregular structure that is broken off in different directions. Figure 3 also indicates that the WSe_2_ nanowires extensively grow on the surface of the graphene nanosheet. Figure 3(c) depicts bright nanowire WSe_2_ shapes that are properly coated on the graphene surface, indicating that graphene nanosheets provide a good platform for the nucleation and successive growth of WSe_2_ layers, and growth on WSe_2_ in graphene is possible due to the precursors that are attached to the graphene oxide through functional groups ^23, 34–36^. Figure 4(a–d) shows transmission electron microscopy (TEM) that further confirms the morphology and shape of the WSe_2_-graphene nanocomposites. Figure 4(a–c) shows the WSe_2_-graphene nanocomposites at different magnifications. The WSe_2_ is clearly seen to have dark imaged compounds that are almost very small in a spherical form, layer form, and a highly agglomerated structure attached to the surface of the graphene sheets. Moreover, several layers of the WSe_2_ are covered with the graphene nano sheets, and the average size of the WSe_2_ is measured to be approximately 6 to 10 um using the ImageJ software.

**Figure 5:**
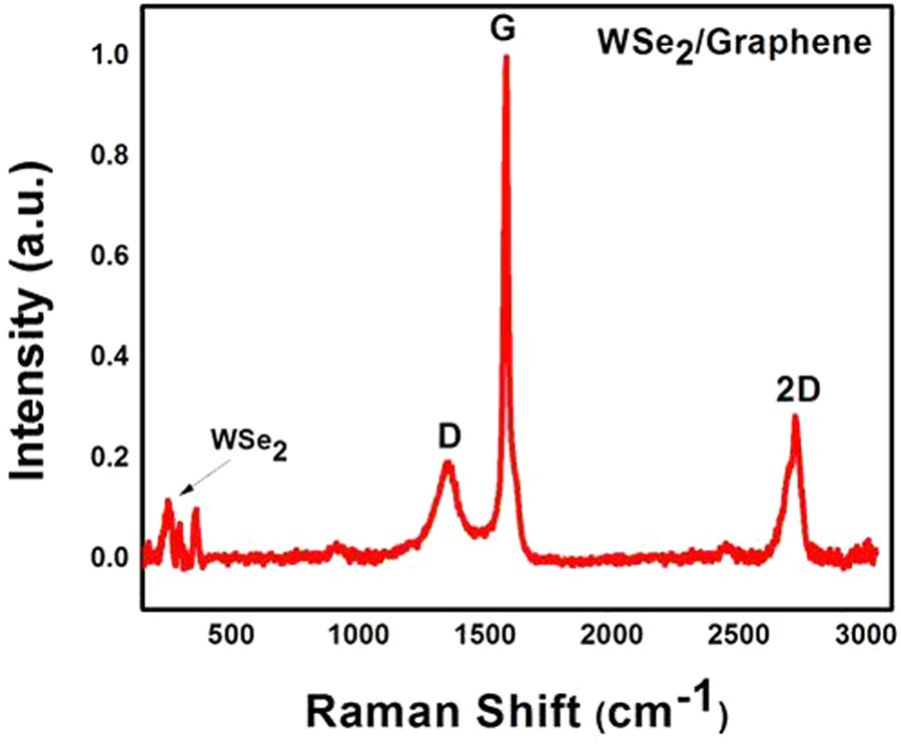
Raman spectra of the WSe_2_-graphene nanocomposite.

Figure 4(d) shows the formation of single-crystalline and few-layered WSe_2_ nanosheets. Different WSe_2_ flakes indicating that the particles sizes fluctuate between 30 nm to 190 nm. When compared to the bulk material, the size of the WSe_2_ layers decreases, which is may be due to the discontinuity of the particles induced by sonication and particle selection by centrifugation^37^. The precise structural properties of the WSe_2_-graphene were analyzed via Raman spectroscopy. Figure 5 provides comprehensive detail of the GO and WSe_2_ nannocomposites (e.g., crystal structure and no. of layers), Fig. 5 shows the Raman signature energy band of the WSe_2_ located at 200 to 400 cm^−1^. The typical Raman spectra for WSe_2_ shows a band at the A_1_g (out-of-plane) (255 cm^−1^) and E_1_2g (inplane) mode (250 cm^−1^)^38^.

Figure 5 also shows that the characteristics of the graphene Raman shifts for the D, G, and 2D bands. The D band is located at 1360 cm^−1^, which shows the presence of disorder in the atomic arrangements or edge effect of graphene, while the G band appears at 1590 cm^−1^. Both the G and D band show the vibration of the carbon atoms in disorder or defect sites and the in-plane vibration of sp2 and sp3 bonded carbon atoms^39^. These two bands (D 1360 cm^−1^, G 1590 cm^−1^) indicate an interaction between rGO and selenide nanosheets, which improves the stability of the catalyst interface and provides a high stability for the nanocomposites. A 2D band appears at 2690 cm^−1^ to express the degree of graphitization. Moreover, the 2D band is smaller than the G band in our spectrum, which indicates the presence of a few layers of graphene sheets^40–43^.

**Figure 6:**
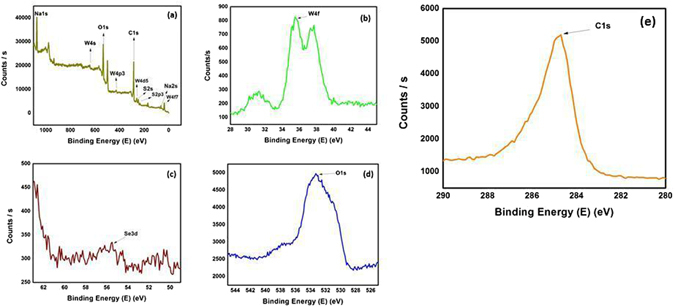
XPS spectra of the WSe_2_-graphene nanocomposite: (**a**) survey scan spectra (**b**) W4f (**c**) Se3d (**d**) O1s and (**e**) C1s.

XPS measurements were carried out to assess the elemental composition of the WSe_2_-graphene nanocomposite. Figure 6(a) shows the XPS spectrum, which indicates the presence of W, Se, C and O and shows the formation WSe_2_-graphene nanocomposite. Figure 6(b) shows the XPS spectrum of W and the binding energies of W4f_7/2_, Wf_5/2_ and W5p_3/2_ at positions of 31.90, 34.80 and 37.90 eV, respectively. The W4f_7/2_ and Wf_5/2_ binding energy peaks express the elemental chemical binding state of W, while the peak positioned at 37.70 eV is attributed to the core level of W5p_3/2_ from WO_3_ due to the partial oxidation of the WSe_2_ layers^44, 45^. The binding energy for the Se 3d_3/2_ core level peak of 54.90 eV confirms a lattice Se^−2^ of the WSe_2_-graphene hybrid (Fig. 6c). The core peak level of Se3d between 50 to 56 eV shows the absorbance of pure Se in a catalysts, which is conclude that WSe_2_-graphene nanocomposite is free from any impurity^46, 47^. Figure 6(d) shows the core level XPS spectrum of the O1s, peak located at 532.90 eV, which shows the carbonyl and carboxyl groups, while the C1s (Fig. 6(e)) spectrum is located at 284.5 eV. These results show that the spectra can fit with oxygen containing functional groups (C-C, C-O), which is evident in the reduction of GO to rGO^48–52^.

**Figure 7:**
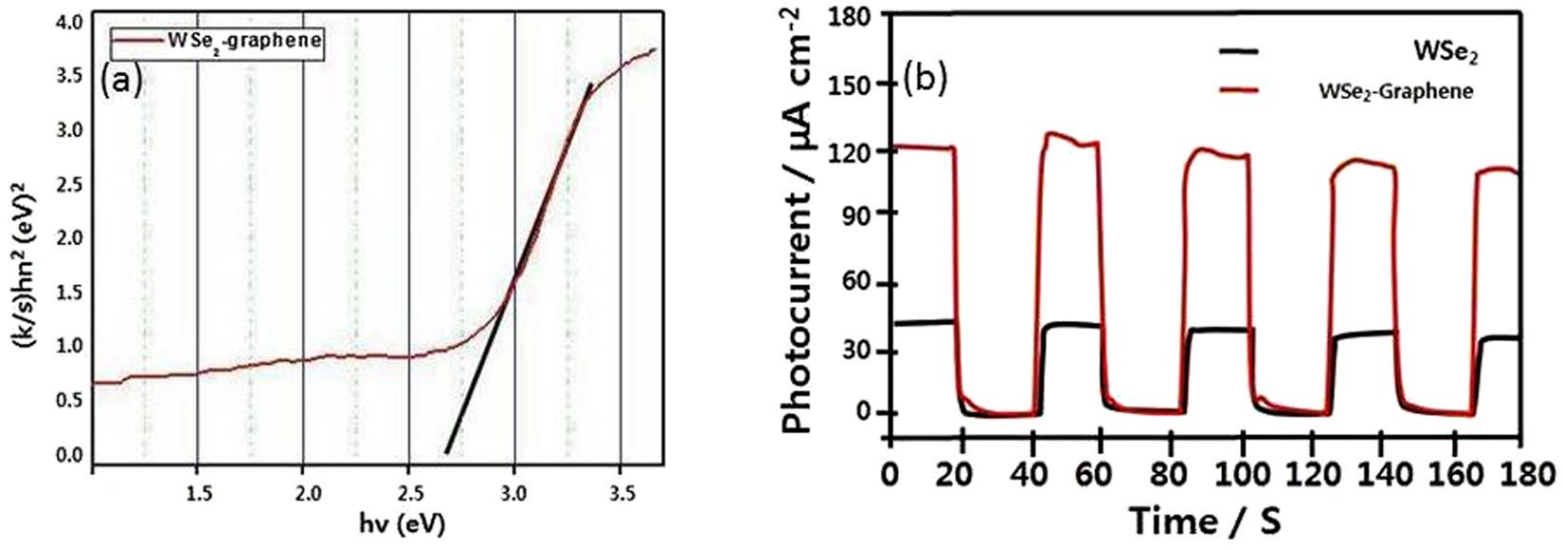
DRS of the WSe_2_-graphene composite and photocurrent measurement with pure WSe_2_ semiconductor (**a**) and WSe_2_-graphene modified photoelectrodes (**b**).

The UV-vis diffuse reflectance spectrum of the WSe_2_-graphene nanocomposite was displayed in Fig. 7(a). The band gap energy value was achieved where the straight line approaching the curve intersects the horizontal axis. As the results from Fig. 7(a), the band gap energy values of the WSe_2_-graphene nanocomposite was 2.68 eV. The photoelectrochemical current response was investigated by employing different materials to decorate the ITO sheet used as a photoanode. The photocurrent of each prepared sample was calculated in a 0.1 M KCl solution containing 0.1 M TEA under “on-off” light illumination cycles at a bias of 0 V vs. Ag/AgCl, as shown in Fig. 7(b). The photoresponse for graphene was non-existent because graphene could not be excited, as shown in curve a^53^ to the nature of graphene, which requires further reduction steps to generate a photocurrent signal^54^. This Fig. 7(b) displays the photocurrent response of the ITO/WSe_2_-graphene based on time, which was repeatedly measured five times at 20-s intervals under visible irradiation. No current was observed in the dark, which clearly suggests that no photoinduced charge separation took place. When the light was turned on, the photocurrent intensity was significantly increased to 121.21 μA. This may have been due to the photoinduced electron–hole separation at the WSe_2_, in which the holes were scavenged by the TEA, and graphene acted as an electron transfer medium. Thus, the electrons were transported to the ITO electrode, resulting in photocurrent generation. The electrode showed a pronounced and stable photocurrent response during the light “on-off” condition, which it maintained.

### Photocatalytic performance

**Figure 8:**
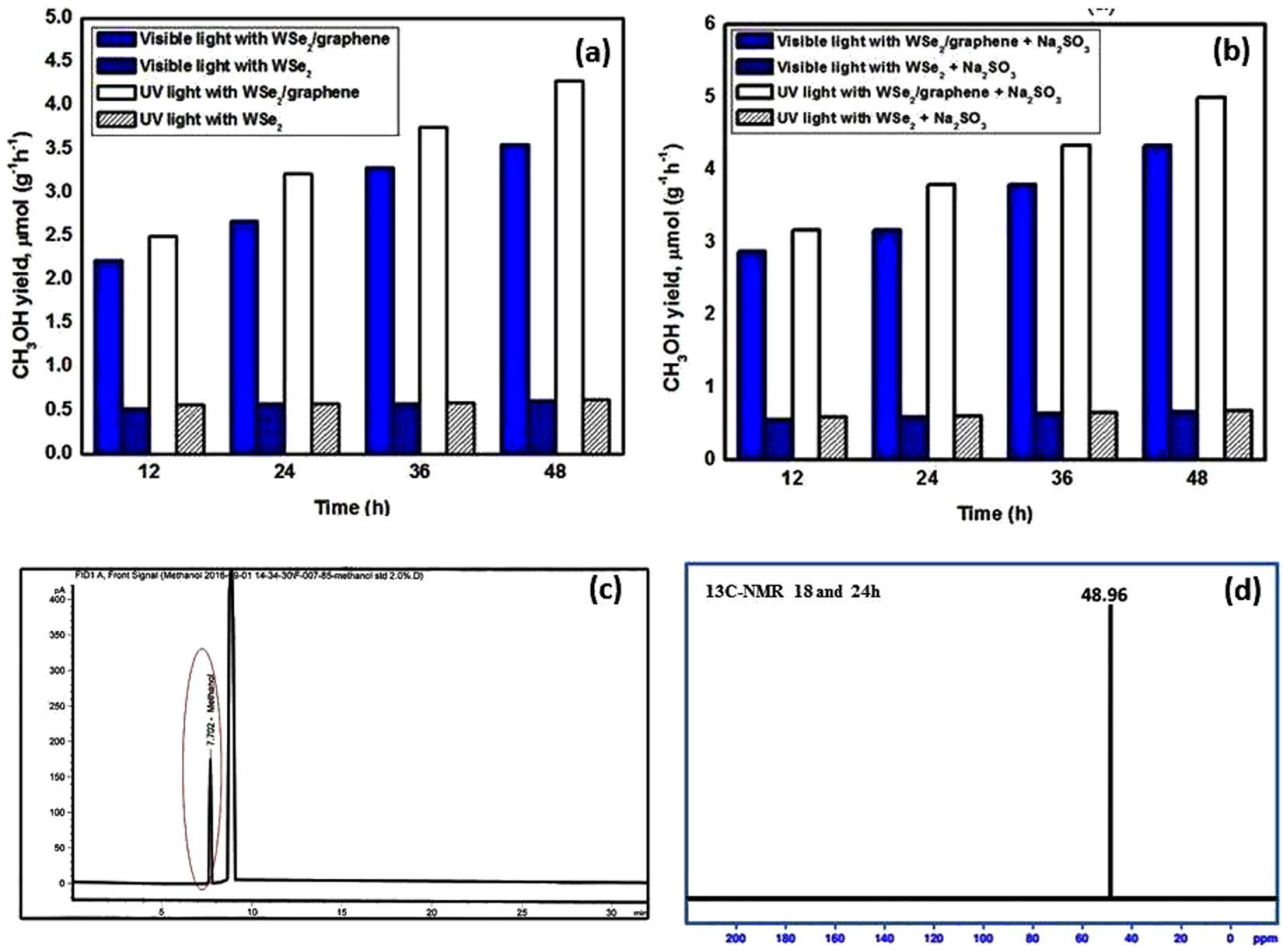
The methanol yields in the photocatalytic reduction of CO_2_ under UV/visible light irradiation by using pure WSe_2_ and WSe_2_-graphene nanocomposits as phtocatalyst (**a**) without Na_2_SO_3_, (**b**) with Na_2_SO_3_ (**c**) GC chromatogram of photoreaction using WSe_2_-graphene, after 48 h and (d) 13 C NMR (Proton decoupled).

We assessed the photocatalytic activity of the pure WSe_2_ and WSe_2_-graphene composites for 48 h during UV (λ > 300) and visible (λ > 400) light irradiation, and every 12 h interval sample withdraw manually from the reactor using a gas-tight syringe, then the collected sample was centrifuged and characterized via gas chromatography. Figure 8 displays the CH_3_OH yield for the pure WSe_2_ and WSe_2_-g nanocomposites. The control experiment determined that no methanol yield was produced in the absence of photocatalysts or light irradiations or both. The gas chromatographic study shows that only CH_3_OH was successfully achieved as a reduction product. Furthermore, graphene is an electron donor for the reduction of CO_2_ as a substitute carbon source to produce CH_3_OH, and H_2_O was a reactant of the CO_2_ reduction. Figure 8(a) shows that the effect of the photocatalytic reduction of CO_2_ with pure WSe_2_ and WSe_2_-graphene under UV light was higher than that for the same composite under visible light.

The methanol yields and quantum yields for the WSe_2_-graphene nanocomposite with different time intervals and different conditions under UV/Visible light irradiation are shown in Tables 1 and 2. The methanol yield and quantum yield with WSe_2_-graphene (12 h), WSe_2_-graphene (24 h), WSe_2_-graphene (36 h) and WSe_2_-graphene (28 h) under visible light were 2.2434 (0.3285), 2.7021 (0.4435), 3.3243 (0.5346) and 3.5509 (0.5779) µmol g^−1^h^−1^, and under UV light were 2.8854 (0.4596), 3.1835 (0.3165), 3.7778 (0.6040) and 4.3257 (0.7053) µmol g^−1^h^−1^, respectively. Na_2_SO_3_ was used as a sacrificial reagent to further enhance the catalytic activity of the binary graphene-based nanocomposites in the photoreduction of CO_2_, as shown in Fig. 8. Figure 8(b) shows that the efficiency of the methanol yield of the pure WSe_2_ and WSe_2_-graphene nanocomposite under UV/visible light is almost two times greater than that of the nanocomposite without using scavenger (Na_2_SO_3_), and Fig. 8(c) shows a GC calibration curve for the quantification of methanol after 48 hours. The sacrificial reagent plays a crucial role in attaining the stability of the photocatalysts because of the well-known process of photocorrosion of sulfides. For further confirmation of final product CH_3_OH, the products were recovered after 18 h and 24 h of irradiation and analyzed by 13 C NMR (Proton decoupled) (Fig. 8d). A single peak was obtained at 48.96 ppm for both, as it is due to methanol, it is verified that photocatalytic conversions of CO_2_ yield mainly CH_3_OH. It is also substantiated by GC. For stability and recyclability, the WSe_2_-graphene nanocomposite was tested for photocatalytic conversion of CO_2_ into CH_3_OH under UV/Visible light irradiation. The WSe_2_-graphene (48 h) was reused for six consecutive runs, and only a minor change in the CH_3_OH yield rate was found, indicating that the prepared nanocomposite highly stable and can be used for a continuous photocatalytic reduction system of CO_2_.

**Table 1:** Methanol Production condition with methanol yields and quantum yields under visible light.

WSe_2_-graphene (visible light)	CH_3_OH yields (µmol g^−1^ h^−1^)	Quantum yields (QE)
WSe_2_- graphene (12 h)	2.2434	0.3285
WSe_2_- graphene (24 h)	2.7021	0.4435
WSe_2_- graphene (36 h)	3.3243	0.5346
WSe_2_- graphene (48 h)	3.5509	0.5779
**WSe**_**2**_ **- graphene** + **Na**_**2**_ **SO**_**3**_ **(visible light)**	**CH**_**3**_ **OH yields (µmol g** ^**−1**^ **h** ^**−1**^ **)**	**Quantum yields (QE)**
WSe_2_- graphene (12 h)	2.8854	0.4596
WSe_2_- graphene (24 h)	3.1835	0.3165
WSe_2_- graphene (36 h)	3.7778	0.6040
WSe_2_- graphene (48 h)	4.3257	0.7053

**Table 2:** Effect of preparation method on methanol yields and quantum yields under Uv light.

WSe_2_- graphene (UV light)	CH_3_OH yields (µmol g^−1^ h^−1^)	Quantum yields (QE)
WSe_2_- graphene (12 h)	2.5134	0.3776
WSe_2_- graphene (24 h)	3.2686	0.4294
WSe_2_- graphene (36 h)	3.7746	0.5672
WSe_2_- graphene (48 h)	2.3523	0.3535
**WSe**_**2**_ **- graphene + Na**_**2**_ **SO**_**3**_ **(UV light)**	**CH**_**3**_ **OH yields (µmol g** ^**−1**^ **h** ^**−1**^ **)**	**Quantum yields (QE)**
WSe_2_- graphene (12 h)	3.1848	0.4115
WSe_2_ - graphene (24 h)	3.7765	0.5606
WSe_2_- graphene (36 h)	4.3246	0.6530
WSe_2_- graphene (48 h)	5.0278	0.8159

**Figure 9:**
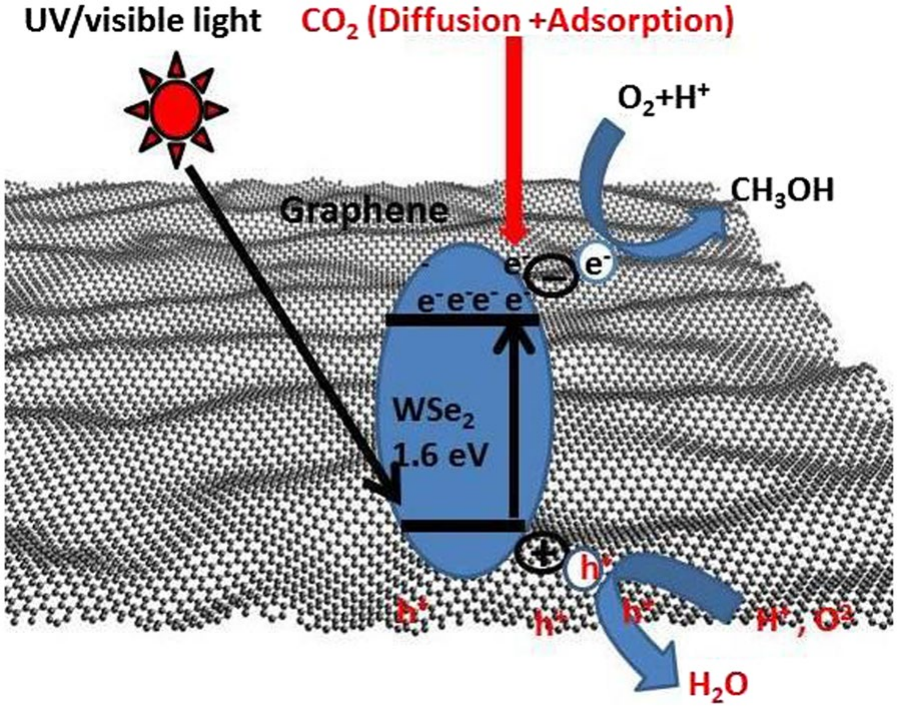
Mechanism study of photocatalytic reduction of CO_2_.

A further explanation of the proposed photocatalytic mechanism is given in Fig. 9, which shows that the WSe_2_ nanomaterial absorbs light of the solar spectrum and creates photo-generated charge carriers (holes and electrons). However, due to the narrow bandgap of the WSe_2_ nanocomposite, these electron-holes recombine very quickly, and their photocatalytic efficiency is therefore limited. To improve the photocatalytic efficiency, the WSe_2_ nanocomposite was attached to a graphene nanosheet since graphene is an electron accepter/transporter that plays an important role in the separation of the transport electron-hole pairs in the binary system^55^. The excited electron and hole in the conduction band of WSe_2_ can be conveniently shifted to the graphene nanosheet, which decelerates the recombination of the electron-hole pairs and thus promotes the electron transport to the catalytic sites for the photo reduction of CO_2_. The graphene with a large surface area and many defective sites absorbs the CO_2_, and the photo-generated electrons on the WSe_2_ are transmitted to the catalytic sites of the graphene and then reduce the absorbed CO_2_ into CH_3_OH^2, 56^. However, the photocatalytic reduction mechanism contains a series of water oxidation and the reduction processes, as shown in Fig. 9. The photo-induced holes on the WSe_2_-g VB could absorb water molecules to form hydroxyl radicals (OH^**·**^), and then the hydroxyl radicals further oxidize the protons (H^+^) and oxygen. In the meantime, electrons in the conductor band transfer and absorb CO_2_ to form ^**·**^CO^−^_2_. The ^**·**^CO_−2_ reacts with **·**H radical, which then leads to the formation of a series of radicals, finally producing CH_3_OH.

Reaction Mechanism.

$$\begin{matrix}{{{\rm{WSe}}}_{2}}^{{\boldsymbol{+}}}\,{\rm{hv}}\to {{\rm{WSe}}}_{{\rm{2}}}({{\rm{e}}}^{{\boldsymbol{-}}}+{{\rm{h}}}^{{\boldsymbol{+}}})\\ {{\rm{WSe}}}_{2}\,({{\rm{h}}}^{+}){\boldsymbol{+}}{{\rm{H}}}_{2}{\rm{O}}\to {{\rm{H}}}^{+}+O\dot{{\rm{H}}}\\ {\rm{Graphene}}\,{(e}^{-}{)+O}_{{\rm{2}}}\to {{{\rm{O}}}_{{\rm{2}}}}^{\cdot }\\ {\rm{Graphene}}\,{(\mathrm{2e}}^{-}{)+\mathrm{2H}}^{+}{{\rm{O}}}_{{\rm{2}}}\to {{\rm{H}}}_{{\rm{2}}}{{\rm{O}}}_{{\rm{2}}}\\ {{\rm{H}}}_{{\rm{2}}}{{\rm{O}}}_{{\rm{2}}}+{\rm{Graphene}}\,{(e}^{-})\to O\dot{{\rm{H}}}\end{matrix}$$

CO_2_ reaction.

$$\begin{matrix}2{{{\rm{CH}}}_{3}}^{+}{\rm{OH}}+3{{\rm{O}}}_{2}\to 2{{\rm{CO}}}_{2}+4{{\rm{H}}}_{2}{\rm{O}}\\ 2{{\rm{CO}}}_{3}+{{\rm{H}}}_{{\rm{2}}}{\rm{O}}+2{{\rm{e}}}^{-}\to {{{\rm{HCO}}}_{2}}^{-}+{{{\rm{HCO}}}_{3}}^{-}\end{matrix}$$

## Methods

### Experimental setup and Materials

Tungsten (VI) oxide (WO_3_), selenium powder (Se, 99%), ammonium hydroxide (NH_4_OH, 25–28%), Sodium sulfite (Na_2_SO_3_ ·7H_2_O, 95%), Nitric acid (HNO_3_) and ethyl alcohol (94%) were purchased from Duksan Pure Chemical Co. Ltd., Korea. All chemicals were used without further purification, and all dilutions were carried out using distilled water.

### Preparation of Graphene

Graphene oxide was prepared in the laboratory following Hummer-Offeman’s method, as previously reported in the literature^57, 58^. A typical preparation method for graphene is as follows. First, 20 g of natural graphite and H_2_SO_4_ (450 ml) are put in de-ionized (DI) water and are stirred continuously for one hour at 0 C. After that, 45 g of KMnO_4_ are slowly mixed with the solution (graphite + H_2_SO_4_) and are constantly stirred at a temperature of 35 °C until it becomes a dim brownish color. Then, the container is sealed and kept at 100 °C with vigorous stirring for 30 min. Meanwhile 20% H_2_O_2_ is added drop wise within 5 min. After that, the solution is washed with acetone and 10% HCl several times to remove the residual metal ions. The solution was then heat-treated in a dry oven at 90 °C for 12 h to obtain the graphite oxide power, then 250 mg graphite oxide power were added to 200 ml DI water, were vigorously stirred for 30 min, and were then ultrasonicated (using Ultrasonic Processor, VCX 750) for 2 h. Finally, the resulting solution was refined and washed several times with hot water and kept in a dry oven for 6 h to obtain the graphene oxide powder.

### Preparation of the WSe_2_ composite

In a typical synthesis process, 0.675 g tungsten (vi) oxide (WO_3_) are dispersed in deionized water, 0.5 M nitric acid are then added drop wise in a three-necked flask (100 mL), and the mixture is heated to 120 °C to eliminate H_2_O and O_2_. In a separate flask, 1.5 g of anhydrous sodium sulfite (Na_2_SO_3_) and 0.3 g crude selenium (Se) powder were dispersed in 200 ml of ethylene glycol with continuous magnetic stirring at 80 °C until a selenium salt was obtained. In the next step, both solutions are transferred to a stainless steel autoclave with a Teflon liner with 20 mL capacity for 24 h at 250 °C in an electric furnace. Finally, the WSe_2_ precipitates are cooled to room temperature, the prepared solution is filtered using 47-mm Whatman filter paper, and the remaining material is heated to a temperature of 350 K for 12 h to obtain a WSe_2_ power.

### Preparation of WSe_2_-graphene nanocomposite

Graphene oxide (200 mg) was added in 150 ml ethylene glycol and was then exfoliated to generate a graphene oxide nanosheet (GONS) dispersion solution via ultrasonication for 30 min. The WSe_2_ powder from the above solution is mixed at equal volumetric ratios of 1:1, and the mixture is sonicated at room temperature for 6 h using a controllable serial-ultrasonic apparatus (Ultrasonic Processor, VCX 750, 500 Watt, Korea, Power 500 Watt, frequency 20 KHz, Amplitude 50%, low intensity). The reaction solution was allowed to cool and settle at room temperature after filtering with 47-mm Whatman filter paper with a pore size of 0.7 mm. The resulting powder was washed with distilled water multiple times and was dried in a vacuum oven at 80 °C for 12 h before heat treatment at 500 °C for 1 h with (Ar) inert atmosphere. The prepared sample was then labeled as WSe_2_-graphene.

### Characterization

The crystal structure and morphology of the prepared samples were measured using monochromatic high intensity Cu Kα radiation (λ = 1.5406 Å) in XRD (Shimadzu XD-D1), Energy dispersive X-ray spectrometer (EDX) was used to measure the atomic percentage of W, Se, and C elements, SEM (JSM-5600 JEOL, Japan) and TEM (JEOL, JEM-2010, and Japan) observations. X-ray photoelectron spectroscopy (XPS) was performed using a VG Scientific VISACA Lab 2000 device with a monochromatic Mg X-ray radiation source, and the Raman spectra of the prepared samples were observed using a spectrometer (Jasco Model Name NRS-3100) with an excitation laser wavelength of 532.06 nm. For quantitative analysis of the CH_3_OH, a Standard Gas Chromatograph-Mass Spectrometer (GCMS-QP2010 SE) was used with a long column. Photoelectrochemical measurements were performed using a self-made photoelectrochemical system installed a 250-W halogen lamp as the irradiation source. The photocurrent measurement was performed by a computer-controlled Versa-STAT-3 electrochemical analyzer. A WSe_2_-graphene modified photoelectrode with an active area of 1 cm^2^ was used as the working electrode, and a Pt wire and saturated Ag/AgCl were used as the counter and reference electrodes, respectively. All the photocurrent measurements were conducted by dipping the WSe_2_-graphene modified photoelectrode into a mixture of 0.1 M KCl and 0.5 M TEA at a constant potential of 0 V vs. Ag/AgCl.

### Photocatalytic reduction of CO_2_

The reduction of CO_2_ with H_2_O in the photocatalytic experiments was carried in a reactor designed in our laboratory, as shown in Fig. 1. The reactor consists of three parts: (1) light source, (2) closed chamber, (30 cm length × 2.0 diameter), (3) CO_2_ + N_2_ gas (N_2_ gas was used to remove gasses from the reactor). 100 mg of photocatalyst (WSe_2_-graphene) and Na_2_SO_3_ as hole scavenger^59^ were added in 20 ml distilled water containing sodium bicarbonate (NaHCO_3_, 0.04 M) and were constantly stirred for one hour. Ultra-high-purity grade CO_2_ gas was purged through the reactor for 30 min, and then the suspension solution was magnetically stirred and irradiated with visible light using a metal halide lamp (500 W, SOLAREDGE700, Perfect Light, China). The distance between the light source and the photocatalyst remained at 10 cm, and a heat sink was equipped in the left side of the chamber to remove the lamp heat. Furthermore, the temperatures inside the reactor were kept at 283.15 K. The reaction continued up to 48 h, and in every 12 h interval, the reactor was allowed to cool down naturally for CH_3_OH desorption from the catalyst. Then, the reaction product was taken from the reactor for GC analysis (GCMS-QP2010 SE).

The reaction quantum yield (QE) is estimated using the CH_3_OH yield, noting that six electrons are required to reduce CO_2_ to CH_3_OH. The equation is as follow.

$$\begin{matrix}{{\rm{\varphi }}}_{{\rm{Methanol}}}( \% )=100\times (6\,\times \,{\rm{mole}}\,{\rm{of}}\,{{\rm{CH}}}_{{\rm{3}}}{\rm{OH}}\,\mathrm{yield})/({\rm{mole}}\,{\rm{of}}\,{\rm{proton}}\,{\rm{absorbed}}\,{\rm{by}}\,{\rm{catalyte}})\\ \,\,-\,{\rm{Mole}}\,{\rm{of}}\,{\rm{proton}}=({\rm{I}}\times {\rm{S}})/({{\rm{N}}}_{{\rm{A}}}\times {\rm{E}})\end{matrix}$$

where: I is light intensity (0.12 mW cm^−2^)

S is the irradiated area of the reactor (30 cm × 15 cm)

E is the photon energy (4.97 × 10^−19^ J at 400 nm)

NA is Avogadro’s constant (6.02 × 1023 mol^−1^)

## Conclusion

In summary, the TMDC (WSe_2_) materials are attached to the graphene nanosheet via ultra-sonication. The SEM and TEM images of the prepared samples show that the WSe_2_ have nanowire morphology that is uniformly distributed on the graphene sheets, and the average size of the nanowires is verified to be from 30 to 130 nm. The graphene sheet-supported WSe_2_ nanowire resulting from the binary structure present excited charge carriers with the combined effect of WSe_2_ and graphene, improving the recombination time. Raman spectroscopy and XPS measurements show an intimate contact and chemical binding interaction between the WS_2_ and GO. Graphene plays a role as an electron mediator to support a binary system and to help provide stable photocatalyst materials. The CO_2_ photo reduction experiment was carried out to investigate the photo catalytic reduction of CO_2_ with the WSe_2_-G nanocomposite, achieving a maximum photocatalytic efficiency after 48 h. The WSe_2_-graphene with added Na_2_SO_3_ (48 h) showed the highest photocatalytic efficacy and obtained a total CH_3_OH yield of 5.0278 µmol g^−1^h^−1^. Our present work indicates that the attachment of WSe_2_ on the graphene sheet can further increase the photocatalytic performance, opening new ways to deploy novel, next-generation heterojunction photocatalysts for environmentally-related applications.
